# Challenges and opportunities facing game theory and control: an interview with Tamer Başar

**DOI:** 10.1093/nsr/nwz154

**Published:** 2019-10-24

**Authors:** By Ji-Feng Zhang

## Abstract

The Control community has recently witnessed an almost exponentially growing interest in the application of game-theoretic concepts and tools in research on control, multi-agent systems, and networks. In an interview with NSR, Professor Tamer Başar, a member of the US National Academy of Engineering, Swanlund Endowed Chair and CAS Professor of Electrical and Computer Engineering, and Director of the Center for Advanced Study at the University of Illinois at Urbana-Champaign in the USA, former president of both the IEEE Control Systems Society and the American Automatic Control Council, and the founding president of the International Society of Dynamic Games, talked about the recently emerging role of game theory in control and networking research, how it broadens the territorial boundaries of control into disciplines outside engineering, and opportunities and challenges that lie ahead.

## WHAT GAME THEORY IS AND ITS ROLE IN CONTROL

**NSR:** Could you tell us briefly what game theory is and what it deals with?

**Başar:** *Game Theory* deals with strategic interactions among multiple decision makers, called *players* (and in some contexts, *agents*), with each player's preference ordering among multiple alternatives captured in an objective function for that player, which she either tries to maximize (in which case the objective function is a *utility* function or a *benefit* function) or minimize (in which case we refer to the objective function as a *cost* function or a *loss* function). For a non-trivial game, the objective function of a player depends on the choices (*actions*, or equivalently *decision variables*) of at least one other player, and generally of all players, and hence a player cannot simply optimize her own objective function independent of the choices of the other players. This thus brings in a coupling among the actions of the players, and binds them together in the decision-making process even in a non-cooperative environment. If the players are able to enter into a cooperative agreement so that the selections of actions or decisions are done collectively and with full trust, so that all players would benefit to the extent possible, then we would be in the realm of *cooperative game theory*, where issues such as bargaining and characterization of *fair* outcomes, coalition formation, and excess utility distribution are of relevance.

If no cooperation is allowed among the players, then we are in the realm of *non-cooperative game theory*, where, for a systematic approach, first one has to introduce a satisfactory solution concept. Leaving aside for the moment the issue of how the players can reach such a solution point, let us think about what minimum feature one would expect such a solution concept to deliver. To first order, the solution point should have the property that if all players but one stays put, then the player who has the option of moving away from the solution point should not have any incentive to do so because she cannot improve her payoff. Note that we cannot allow two or more players to move collectively from the solution point, because such a collective move requires cooperation, which is not allowed in a non-cooperative game. Such a solution point where none of the players can improve their payoffs through unilateral moves is known as a *non-cooperative equilibrium* or *Nash equilibrium*, named after John Nash, who introduced it and proved that it exists in finite games (that is, games where each player has only a finite number of alternatives) and in mixed strategies, over 60 years ago. Another non-cooperative equilibrium-solution concept is the *Stackelberg equilibrium*, which in fact predates the Nash equilibrium, where there is a hierarchy in decision-making among the players, with some of the players, designated as *leaders*, having the ability to first announce their actions (and make a commitment to play them), and the remaining players, designated as *followers*, taking these actions as given in the process of computation of their non-cooperative (Nash) equilibria (among themselves). Before announcing their actions, however, the leaders would of course anticipate these responses and determine their actions in such a way that the final outcome will be most favorable to them (in terms of their objective functions).

**Figure:**
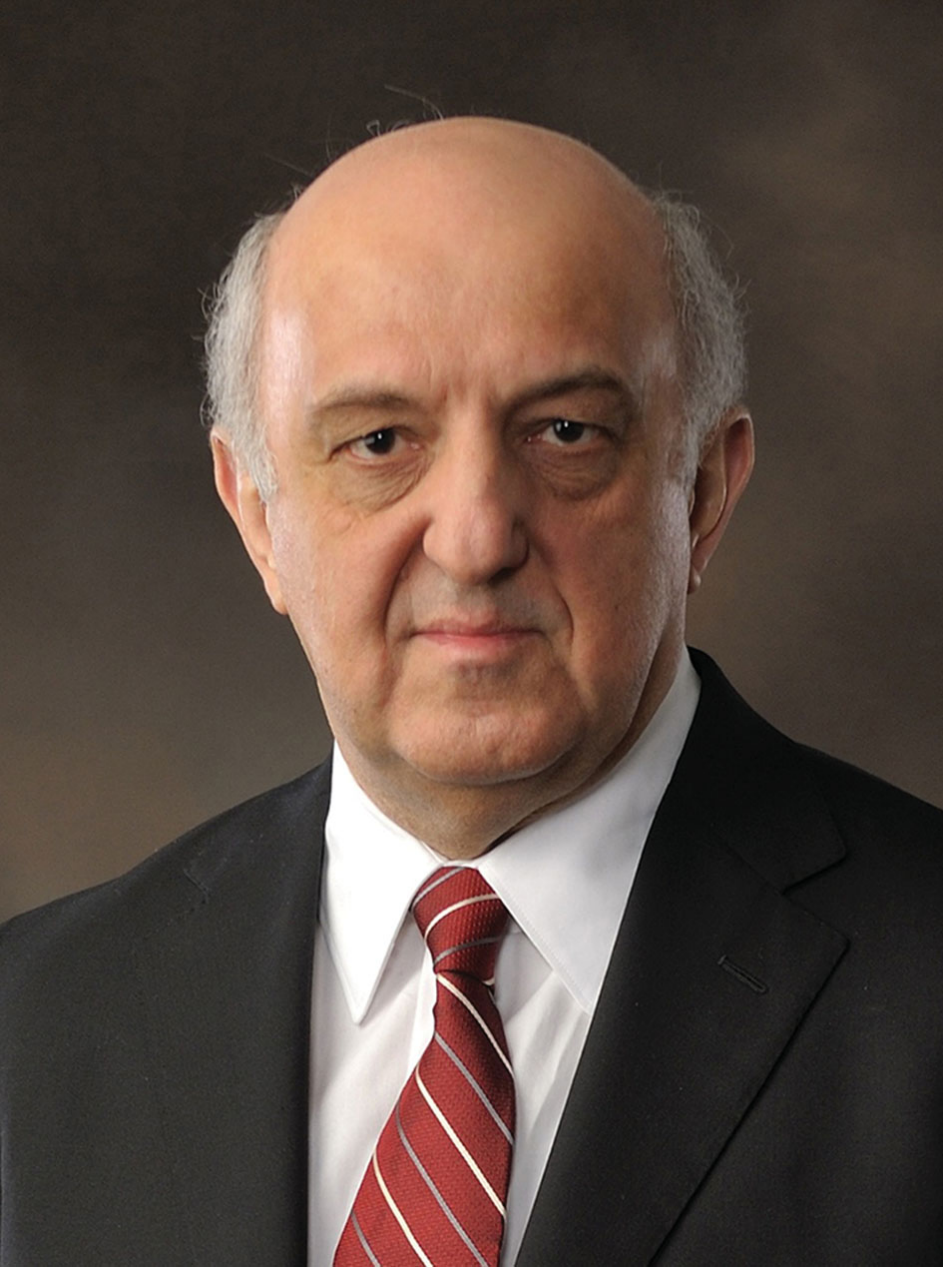
Tamer Başar, Swanlund Endowed Chair and CAS Professor of Electrical and Computer Engineering, and Director of the Center for Advanced Study at the University of Illinois at Urbana-Champaign *(Courtesy of Tamer Başar)*.

**NSR:** What are the different types of games we encounter?

**Başar:** We can place games into two broad categories, as cooperative and non-cooperative games. For the latter class, which is of more interest to the broader control community, we can have a further sub-classification. We say that a non-cooperative game is strictly (or genuinely) *nonzero-sum*, if the sum of the players' objective functions cannot be made zero after appropriate positive scaling and/or translation that do not depend on the players' decision variables. If a game has only two players, we say that it is *zero-sum* if the sum of the objective functions of the two players is *zero* or can be made zero by appropriate positive scaling and/or translation that do not depend on the decision variables of the players. On a broader scale (and with a possible abuse of terminology), we can view two-player zero-sum games as constituting a special sub-class of two-player nonzero-sum games, and in this case the Nash equilibrium becomes the *saddle-point equilibrium*.

A further sub-classification is according to the nature of the action alternatives a player has access to and how these actions determine the outcome. A game is a *finite game* if each player has only a finite number of alternatives, that is the players pick their actions from finite sets (action sets); otherwise the game is an *infinite game*; finite games are also known as *matrix games*. An infinite game is said to be a *continuous-kernel game* if the action sets of the players are continua and the players' objective functions are continuous with respect to action variables of all players. A game is said to be *deterministic* if the players' actions uniquely determine the outcome, as captured in the objective functions, whereas if the objective function of at least one player depends on an additional variable (state of nature) with an underlying probability distribution, then we have a *stochastic game*. A game is a *complete information* game if the description of the game (that is, the players, the objective functions, and the underlying probability distributions (if stochastic)) is common information to all players; otherwise we have an *incomplete information* game. We say that a game is *static* if players have access to only the *a priori* information (shared by all), and none of the players have access to information on the actions of any of the other players; otherwise what we have is a *dynamic game*. A game is a *single-act game* if every player acts only once; otherwise the game is *multi-act*. Note that it is possible for a single-act game to be dynamic and for a multi-act game to be static. A dynamic game is said to be a *differential game* if the evolution of the decision process (controlled by the players over time) takes place in continuous time and generally involves a differential equation; if it takes place over a discrete-time horizon, the dynamic game is sometimes called a *discrete-time game*.

**NSR:** The notion of a *strategy* plays an important role in games, particularly if they are *dynamic*, isn’t it?

**Başar:** Indeed. In dynamic games, as the game progresses players acquire information (complete or partial) on past actions of other players, and use this information in the selection of their own actions (also dictated by the equilibrium solution concept at hand). In finite dynamic games, for example, the progression of a game involves a *tree structure* (also called *extensive form*) where each node is identified with a player along with the time when she acts, and branches emanating from a node show the possible moves of that particular player. A player, at any point in time, could generally be at more than one node, which is a situation that arises when the player does not have complete information on the past moves of other players, and hence may not know with certainty which particular node she is at, at any particular time. This uncertainty leads to a clustering of nodes into what are called *information sets* for that player. What players decide on within the framework of the extensive form is not their actions, but their *strategies*, that is what action they would take at each information set (in other words, correspondences between their information sets and their allowable actions). They then take specific actions (or actions are executed on their behalf), dictated by the strategies chosen as well as the progression of the game (decision) process along the tree. The equilibrium is then defined in terms of not actions but strategies. If I have to draw a parallel with control theory, strategies are like *control laws*, or equivalently *control policies*, which act on the information available to the controller, generally regarding the state of the system being controlled, which may be acquired through perfect or noisy channels, to generate control signals (or commands) as inputs to the system. Two extreme cases in a control system scenario would be the case with absolutely no state information, which corresponds to *open-loop control*, and the case of full state information, which corresponds to *state-feedback control*. In dynamic games, these two extreme cases are of course also present, but the possibility of different players having access to different information (such as, some open-loop and some full-state) makes the landscape much more complex.

**NSR:** This may now be the right point to ask whether there is any connection between control and dynamic games?

**Başar:** Indeed there is. Control, particularly optimal control, can be viewed as a one-player dynamic (or differential) game.

In dynamic games…the possibility of different players having access to different information…makes the landscape much more complex.

—Tamer Başar

Further, since characterization and computation of Nash equilibrium involve solutions of individual optimization problems for each player, tools developed for optimal control, such as dynamic programming or maximum principle, would definitely be useful in dynamic games. However, as I mentioned earlier, the situation is more complex for dynamic games if players have different types of information, and even if these are of the type open-loop for some players and full-state for others neither dynamic programming nor maximum principle could be used in the construction of Nash equilibria. We know that in deterministic optimal control it is possible to synthesize optimal state-feedback controllers from optimal open-loop controls (using the corresponding state trajectory); this, however, is no longer the case with deterministic nonzero-sum dynamic games, because of the strategic interaction that exists among the players and the fact that Nash equilibria under open-loop and state-feedback strategies do not lead to the same outcomes for the players. With other types of information structures, which may also incorporate partial information on the past actions of the players, or for stochastic dynamic games, the situation is even more complex, and tools to be employed for characterization or computation of equilibria are very much information structure dependent. This is still an active area of research, and intricacy of information structures in dynamic games is a topic that has been of major interest to me for many years and continues to be.

For zero-sum dynamic or differential games, the situation is more tractable, and that has led to success stories in robust control where the system may have modeling uncertainties and/or unknown inputs. The zero-sum game-theoretic approach to robust control allows the *unknowns* to be treated as inputs controlled by an adversary who has an objective completely opposite of that of the controller. This direct conflict of interest between the controller and a fictitious adversary leads to a zero-sum dynamic game formulation, whose minimax or saddle-point solution under a given information structure for the controller, which is also shared by the adversary (who is the maximizing player), leads to a robust control law for the system. This approach has led to optimal H-infinity designs for linear as well as nonlinear systems under different information structures, and has also been adopted by economists, such as the Nobel Laureates Lars Hansen and Thomas Sargent in their 2008 Princeton University Press book *Robustness*.

## HISTORICAL EVOLUTION OF GAME THEORY

**NSR:** How far back can we trace the origins of game theory? Could you give us a little bit of the history?

**Başar:** Kick-start of the field is generally acknowledged to be the publication of the book *Theory of Games and Economic Behavior* by John von Neumann and Oskar Morgenstern in 1944. Since then, game theory has enjoyed incessant growth in both the number of theoretical results and the scope and variety of applications. As a recognition of the vitality of the field, up to this point a total of 10 Nobel Prizes have been awarded in Economic Sciences for work primarily in game theory, with the first recognition bestowed in 1994 on John Harsanyi, John Nash, and Reinhard Selten ‘for their pioneering analysis of equilibria in the theory of non-cooperative games’. The second round of Nobel Prizes in game theory went to Robert Aumann and Thomas Schelling in 2005, ‘for having enhanced our understanding of conflict and cooperation through game-theory analysis’. The third round recognized Leonid Hurwicz, Eric Maskin, and Roger Myerson in 2007, ‘for having laid the foundations of mechanism design theory’. And the most recent one was in 2012, recognizing Alvin Roth and Lloyd Shapley, ‘for the theory of stable allocations and the practice of market design’. To this list of highest-level awards related to contributions to game theory, I should also add the 1999 Crafoord Prize (which is the highest prize in Biological Sciences), which went to John Maynard Smith (along with Ernst Mayr and G. Williams) ‘for developing the concept of evolutionary biology’, where Smith's recognized contributions had a strong game-theoretic underpinning, through his work on evolutionary games and evolutionary stable equilibrium.

Even though von Neumann and Morgenstern's 1944 book is generally taken as the starting point of the scientific approach to game theory, game-theoretic notions and some isolated key results date back to earlier years and even centuries. Sixteen years earlier, in 1928, John von Neumann himself had resolved completely an open fundamental problem in zero-sum games, that *every finite two-player zero-sum game admits a saddle point in mixed strategies*, which is known as the *Minimax Theorem*—a result which Emile Borel had conjectured to be false eight years before. Some early traces of game-theoretic thinking can be seen in the 1802 work (*Considérations sur la Théorie Mathématique du Jeu*) of André-Marie Ampère (1775–1836), who was influenced by the 1777 writings (*Essai d’Arithmétique Morale*) of Georges Louis Buffon (1707–1788).

The zero-sum game-theoretic approach to robust control allows the *unknowns* to be treated as inputs controlled by an adversary who has an objective completely opposite of that of the controller.

—Tamer Başar

Since then [1944], game theory has enjoyed incessant growth in both the number of theoretical results and the scope and variety of applications.

—Tamer Başar

**NSR:** Have there been parallel developments in optimal control during that period, and any impact on the development of differential game theory?

**Başar:** Indeed, in about the same time framework, we see dynamic programming being introduced by Richard Bellman (in the 1950s), while working at the RAND Corporation, as a major principle and tool for optimal control, and multi-stage decision making in a broader sense. In the early 1950s, RAND had attracted and housed some of the greatest minds of the time, among whom were, in addition to Bellman, names like Leonard D. Berkovitz, David Blackwell, George Dantzig, Wendell Fleming, M.R. Hestenes, Rufus Isaacs, Samuel Karlin, John Nash (whose name I mentioned earlier), J.P. LaSalle, and Lloyd Shapley (to list just a few). These people, and several others, laid the foundations of decision and game theory, which subsequently fueled the drive for control research. In this unique and highly conducive environment, Bellman actually started working on multi-stage decision processes, as early as 1949, but more fully after 1952—and it is perhaps a lesser known historical fact that one of the earlier topics Bellman worked on at RAND was game theory (both zero- and nonzero-sum games) on which he co-authored research reports with Blackwell and LaSalle. In an informative and entertaining autobiography he wrote 32 years later (*Eye of the Hurricane*, World Scientific, Singapore), completed in 1984 shortly before his untimely death (March 19), Bellman describes eloquently the research environment at RAND and his reason for coining the term ‘dynamic programming’. Applying dynamic programming to different classes of problems, and arriving at ‘functional equations of dynamic programming’ subsequently led Bellman, as a unifying principle, to the ‘Principle of Optimality’, which Isaacs, also at RAND, and at about the same time, had called ‘tenet of transition’ within the broader context of differential games, capturing strategic dynamic decision-making in adversarial environments. Isaacs is in fact credited for coining the word ‘differential games’ to competitive continuous-time dynamic decision-making in a zero-sum framework, and some also give credit to him for the principle of optimality, since it can be viewed as a special case of the tenet of transition. Due to the classified nature of his work, Isaacs was not able to publish his findings in the open literature (as Bellman was able to) for many years, until 1965 when his book *Differential Games* appeared, which generated a quantum jump in research interest, first in pursuit-evasion games (more broadly, zero-sum differential games) and later in the early 1970s in nonzero-sum differential games, spearheaded by Yu-Chi (Larry) Ho at Harvard and his collaborators.

## RESEARCH CHALLENGES IN GAME THEORY

**NSR:** Could you tell us what, at present, the main areas of research in game theory are, particularly in dynamic games, and what the main challenges are?

**Başar:** I’ll mention just a few, which is definitely not a complete exhaustive list. As I briefly mentioned earlier, the role information structures play in both the characterization and computation of non-cooperative equilibria in dynamic games is a currently active area of research, as there are still so many intricacies, which have not yet been fully sorted out. These all involve the dependence of equilibria on the kind of information players acquire during the decision-making process, who communicates with whom, and how actions of a particular player affect the quality and content of relevant information other players receive—information relevant to their decision-making processes. Naturally, none of these issues would arise in single-decision-maker scenarios. There is also the issue of *rationality* of different players—intentional or unintentional loss of rationality—and, even if they are rational to some extent, what would be the ‘bounds’ on their rationality (caused perhaps due to restraints imposed by constraints on resources, such as computing power or sensing capability), and how all this affects equilibria. Yet another issue is that of *robustness* of equilibria to unmodeled uncertainties and adversarial interventions (such as, on communication links), which can, even in a single-decision-maker problem (or somewhat equivalently in control of a deterministic or stochastic system), be cast in a game-theoretic framework, with the unknown uncertainties and/or intrusions on the operation of the system being attributed to a fictitious player whose objective is the complete opposite of that of the decision maker. I had mentioned earlier the success story in connecting robust (H-infinity) optimal control to zero-sum differential games, which had impact in other disciplines as well, and this offers a considerably fertile pathway toward establishing similar connections with nonzero-sum dynamic games.

Another fertile area of research is *network games*. The context here is that, broadly speaking, there is an underlying network or graph structure, possibly multi-layer, that governs the interactions among the players, with neighborhoods determining who communicates with whom, who collaborates with whom, and which players’ objectives or dynamics are coupled with each other. Taking into account such structures in the analysis and computation of equilibria, besides being important in its own right (such as in addressing individual self-interest driven behavior in social networks), would lead to alleviation of some of the complexities I mentioned earlier with regard to the role information structures play in dynamic games. Yet another fertile area of research is *mean-field games*, which involves another type of structural specificity, where a player's interaction is not with other individual players but with an entity corresponding to an infinite population of players, where actions of a single member of that population have only an infinitesimal impact on the emerging behavior of the population. Studying mean-field games, even when the population is not infinite but

…a growing area of current research entails incorporation of tools of machine learning and particularly reinforcement learning into game theory, to handle ‘model-free’ multi-agent decision-making problems.

—Tamer Başar

sufficiently large, alleviates many of the information structural issues I mentioned earlier, as it is the aggregate behavior of the population that matters, and not the individual player behavior that depends strongly on the nature of the available information to that particular player. This is a topical area I myself am currently interested in.

Finally, a growing area of current research entails incorporation of tools of machine learning and particularly reinforcement learning into game theory, to handle ‘model-free’ multi-agent decision-making problems, where players do not have information on the dynamics or objective functions of other players whose actions affect their performance, but incorporate into their action-generating algorithms the data they collect through their observables, such as ‘rewards’ they receive during the decision-making process. NSR had a special topic on ‘Machine Learning’ back in January 2018 (volume 5, issue 1) and I see a big potential in expanding the frameworks and tools presented in that issue to multi-agent systems within a game-theoretic framework, which is one of my current areas of interest.

## HOW TO PREPARE FOR RESEARCH IN GAME THEORY

**NSR:** What would be your advice to a new entrant to the field, such as a graduate student? What background should they acquire before delving into research in game theory, particularly dynamic games?

**Başar:** First, a strong background in mathematics, particularly real analysis, is a must. Second, a strong knowledge of control theory (both deterministic and stochastic, as well as optimal) is essential before confronting the intricacies that arise in going from single to multiple players. Of course, as part of this, solid knowledge of probability and stochastic processes, optimization, and some introductory game theory would be needed. Finally, depending on what specific application area the research to be conducted deals with, some domain knowledge would be essential. It goes, of course, without saying that a new entrant to a research area should familiarize herself/himself with the established as well as the current relevant literature on the topic.

## CONCLUSION

**NSR:** Thank you very much for participating in this interview for this special topic of NSR, and for your insightful comments and outlook on this emerging field.

**Başar:** Thank you for giving me this unique opportunity. I also know that there is strong interest in China in this field, and I see growing activity coming from Chinese researchers, having impact on current developments.

